# A Case of Alcohol Withdrawal-Induced Central and Extrapontine Myelinolysis

**DOI:** 10.7759/cureus.41640

**Published:** 2023-07-10

**Authors:** Maria Jamil, Abdus Salam, Belinda M Joseph Benher, Sheema Rehman, Javairia Jamil, Geehan Suleyman

**Affiliations:** 1 Internal Medicine, Henry Ford Health System, Detroit, USA; 2 Internal Medicine, Khyber Teaching Hospital, Peshawar, PAK; 3 Internal Medicine, Wayne State University, Detroit, USA; 4 Internal Medicine, Henry Ford Health System, Detriot, USA; 5 Internal Medicine, Gulf Medical University, Ajman, ARE; 6 Infectious Disease, Henry Ford Health System, Detroit, USA

**Keywords:** young female patients, alcohol use, extra pontine myelinolysis (epm), central pontine myelinolysis (cpm), osmotic demyelination syndrome (ods)

## Abstract

A 40-year-old female with a history of chronic alcohol use disorder presented with an acute intractable left-sided headache for three days and progressively worsening unsteady gait requiring a wheelchair to ambulate. The patient had a history of chronic alcoholism since 2019 but reported abstinence since September 2021. One month after quitting alcohol, she experienced a sudden deterioration in bilateral extremity neuropathy, forgetfulness, difficulty writing, and severe mood swings, which continued to worsen until her presentation in July 2022. Laboratory tests, including complete blood count and electrolyte levels, were within normal ranges. A previous MRI performed during the investigation for alcoholic neuropathy a few months before she quit drinking showed no abnormalities. However, a subsequent MRI during work-up for the current acute symptoms revealed significant signal abnormalities involving the central pons, bilateral cerebral peduncles, and medullary pyramids, consistent with chronic central pontine myelinolysis (CPM) with extrapontine myelinolysis (EPM) extending into the peduncles. The patient received treatment with folate and multivitamins and was scheduled for outpatient follow-up with physical therapy for rehabilitation. This case highlights CPM as a consequence of alcohol withdrawal and emphasizes the importance of timely diagnosis and appropriate management in such patients.

## Introduction

Osmotic demyelination syndrome (ODS) is a non-inflammatory demyelination disorder that encompasses both extrapontine myelinosis (EPM) and central pontine myelinolysis (CPM). It was initially described in 1959 [1]. ODS manifests with a variety of clinical presentations, including altered consciousness/encephalopathy, dysphagia, and limb weakness [2]. The underlying cause and pathogenesis of ODS remain unclear; however, numerous studies have implicated the rapid correction of hyponatremia as the primary factor associated with CPM, resulting in damage to specific brain regions, particularly the pontine white matter tracts [3]. Although other causes have been suggested, a limited number of cases have reported an association between ODS and alcohol withdrawal. In this article, we present a case of CPM and EPM in a young female who experienced these conditions following the cessation of alcohol use, despite having normal electrolyte levels.

## Case presentation

A 40-year-old female presented at the emergency department with a persistent acute left-sided headache that had been unresponsive to medication for a duration of three days. The headache was sharp and severe accompanied by photophobia and nausea, with no radiation or other eye symptoms. Further history revealed a progressive deterioration of symptoms over the past 10 months, including difficulty writing, increased forgetfulness and gait disturbances. Initially, the patient required a walker for mobility due to lower limb weakness and gait disturbances, but upon admission, she relied on a wheelchair. Her medical history included chronic alcohol use disorder, alcoholic neuropathy, and alcoholic liver cirrhosis. Although she had been a chronic alcoholic since 2019, she reported discontinuing alcohol consumption in September 2021. There had been no hospital admissions since quitting alcohol until her presentation in July 2022. She mentioned an increased appetite after cessation and consumed more than usual for the first few weeks.

Prior to the onset of her current symptoms, she exhibited mild neuropathy in both lower extremities attributed to her alcoholism, characterized by lower limb weakness. However, she reported a sudden worsening of neuropathy in both extremities one week after abruptly stopping her alcohol intake. Over the following months, her symptoms progressed and worsened. During the physical examination, the patient exhibited 3/5 motor strength in her lower extremities, without any concurrent fasciculations or sensory or cranial nerve deficits. However, due to her wheelchair-bound condition, an assessment of her gait could not be performed. Laboratory tests, including complete blood count, creatinine, and electrolyte levels, were all within normal limits, except for a mild decrease in potassium to 3.3, as shown in Table 1.

**Table 1:** Basic metabolic profile BUN: Blood Urea Nitrogen, mEq/L: Milliequivalents Per Liter, mmol/L: Millimoles Per Liter, mg/dl: Milligrams Per Deciliter

Sodium	Potassium	Chloride	BUN	Creatinine	Calcium	Magnesium
138 mEq/L (135-145mEq/L)	3.3 mEq/L (3.5 to 5.2mEq/L)	107 mEq/L (98-106mEq/L)	3 mmol/L (2.1 to 8.5mmol/L)	0.45 mg/dl (0.7 to 1.3mg/dl)	8.9mg/dl (8.5 to 10.2mg/dl)	1.9 mg/dl (1.7 to 2.2mg/dl)

**Figure 1:**
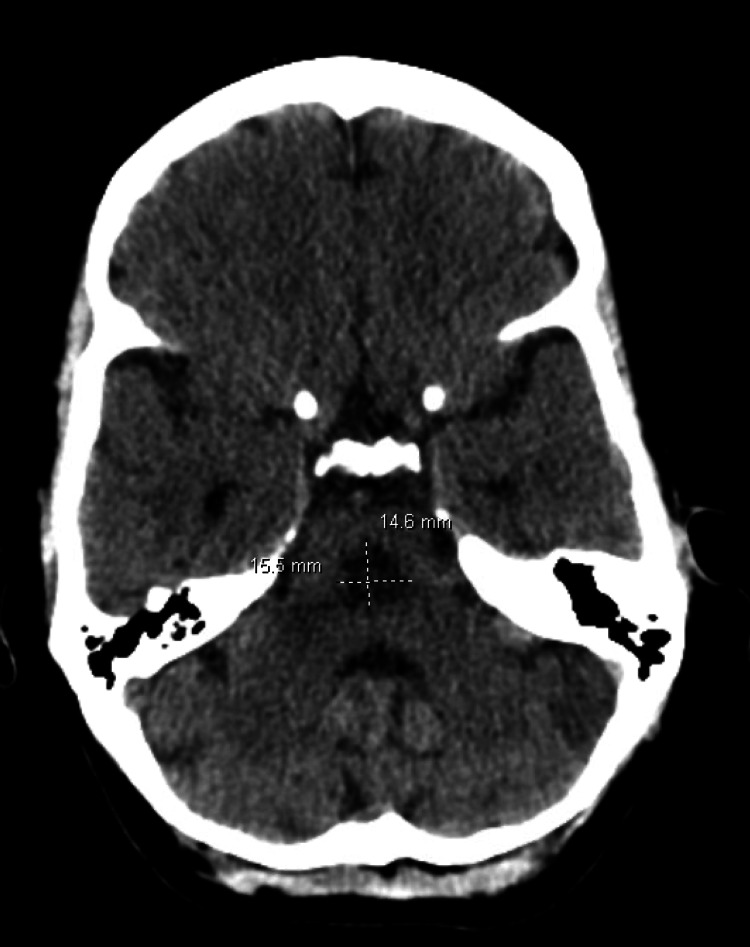
Computed tomography (CT) of the head

A computed tomography (CT) scan of the head (Figure 1) revealed an ill-defined area of reduced density in the central pons measuring 1.5x1.6x1.6 cm.

**Figure 2:**
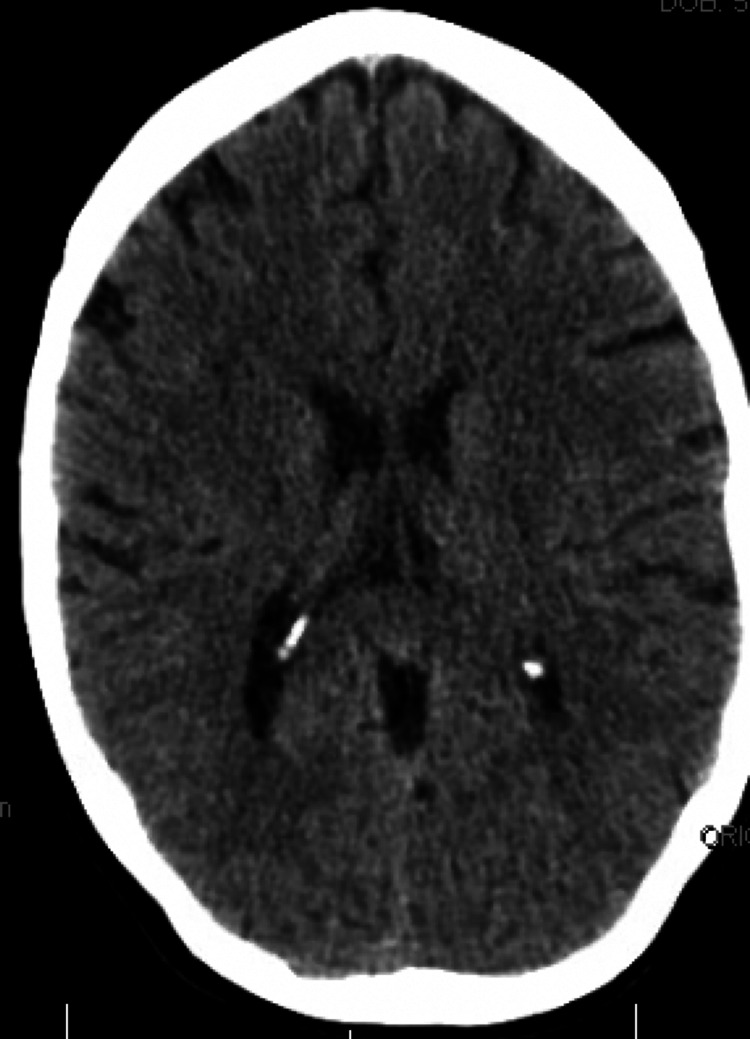
Computed tomography (CT) of the head

There were also scattered areas of reduced density in the subcortical white matter (Figure 2), including the bilateral frontal lobes and right parietal lobe. These CT findings aligned with the clinical presentation of the patient, who exhibited acute intractable left-sided headache, progressive unsteady gait, and other neurological symptoms. The presence of hypoattenuation in the central pons corresponded to the patient's gait disturbances and supports the final diagnosis.

**Figure 3:**
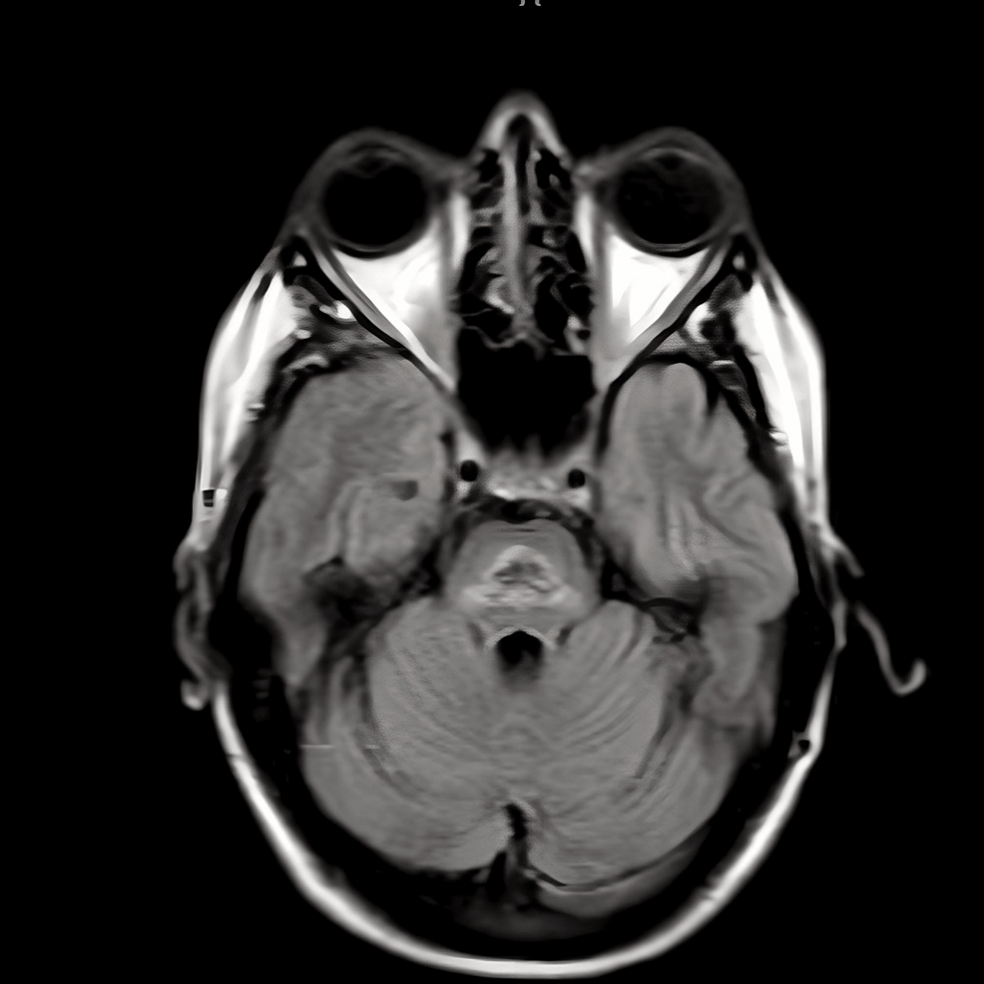
Magnetic resonance imaging of the brain

To further evaluate the condition, a contrast-based magnetic resonance imaging (MRI) scan of the brain was ordered. The MRI (Figure 3) showed heterogenous high T2 signals abnormality with peripheral high FLAIR signals within the central pons, extending into the bilateral cerebral peduncles and the medullary pyramids. These findings strongly indicated chronic central pontine myelinolysis with extrapontine myelinolysis affecting the peduncles, as well as associated Wallerian degeneration in the medulla.

The patient was diagnosed with central pontine myelinolysis and extra pontine myelinolysis resulting from alcohol withdrawal. 

## Discussion

Central pontine myelinolysis (CPM) and extrapontine myelinolysis (EPM) are rare neurological disorders that can occur during alcohol withdrawal. Although the exact pathogenesis of CPM and EPM remains unclear, several studies suggest that cytotoxic edema is a central factor in CPM, while vasogenic edema plays a significant role in EPM [4-5].

CPM is characterized by the loss of myelin in the central region of the basis pons, primarily resulting from the rapid correction of hyponatremia [4-6]. In cases of CPM, the rapid correction of hyponatremia leads to increased extracellular osmolarity, causing water to move from cells into the extracellular space. This change in osmolarity results in a decrease in intracellular osmolarity, which subsequently leads to the development of cytotoxic edema and myelinolysis (4-5). However, it is worth noting that some cases of CPM can occur without hyponatremia [4-6], suggesting that cytotoxic edema plays a more prominent role in these instances [4-5]. Cytotoxic edema occurs when water enters the cells, causing cell swelling and damage. The precise mechanism by which alcohol withdrawal induces cytotoxic edema and CPM is not fully understood, but it is believed to involve changes in the osmotic gradient and the transport of water and electrolytes across the blood-brain barrier [4].

EPM is characterized by the loss of myelin in the brainstem, cerebellum, and other areas outside the pons [4]. It can occur during alcohol withdrawal, even in the absence of hyponatremia [5-7]. Vasogenic edema is considered a significant contributor to the pathogenesis of EPM [4-5]. Vasogenic edema arises from the breakdown of the blood-brain barrier, leading to the accumulation of fluid in the extracellular space. The precise mechanism by which alcohol withdrawal triggers vasogenic edema and EPM is not fully understood, but it is thought to involve changes in the permeability of the blood-brain barrier and the accumulation of toxic substances in the brain [4].

Chronic alcoholism is a recognized risk factor for developing both CPM and EPM [4-5]. Alcohol can induce changes in the blood-brain barrier, resulting in the accumulation of toxic substances in the brain and subsequent demyelination [4]. Moreover, chronic alcoholism can contribute to malnutrition, further increasing the susceptibility to CPM and EPM [4].

Although there have been reports implicating causes other than hyponatremia in the development of CPM and EPM, a few cases have suggested a potential association with alcohol withdrawal and refeeding syndrome [4-7].

Refeeding syndrome is a condition characterized by dangerous shifts in electrolytes and fluids in malnourished patients who receive artificial feeding, either parenterally or enterally. This syndrome can lead to metabolic and hormonal changes that give rise to clinical complications. One of the primary manifestations of refeeding syndrome is the development of hypophosphatemia. Additionally, changes in glucose, thiamine, protein, and fat metabolism can occur, contributing to neurological complications. In the case of our patient, who engaged in binge drinking for the past two years and abruptly ceased in September 2022, she reported a significant increase in appetite and consumed more food than usual in the following week. One week later, she experienced a deterioration in bilateral neuropathy and the onset of weakness and forgetfulness. While no abnormal changes in her electrolytes were observed upon admission, we cannot exclude the possibility of refeeding syndrome as a potential cause of her subsequent development of CPM, considering her presentation to the hospital 10 months later.

The primary treatment for CPM and EPM is supportive care, with the prognosis dependent on the severity of the neurological symptoms [4-6]. Due to their potential for fatal outcomes, it is crucial to promptly consider CPM and EPM when faced with unexplained neurological symptoms during alcohol withdrawal or in patients with a history of chronic alcohol abuse [4-5]. This case highlights the significance of conducting comprehensive diagnostic studies to ensure accurate diagnosis in such cases. Furthermore, an accurate diagnosis of CPM would enable the implementation of appropriate rehabilitation programs, which are essential for patients with CPM secondary to chronic alcoholism.

## Conclusions

It is essential to include CPM and EPM in the differential diagnosis of patients who manifest new onset psychiatric or neurological symptoms after alcohol withdrawal. Further research is necessary to understand the pathophysiology and risk factors for CPM and EPM induced by alcohol withdrawal.
